# Selecting the optimal healthcare centers with a modified P-median model: a visual analytic perspective

**DOI:** 10.1186/1476-072X-13-42

**Published:** 2014-10-22

**Authors:** Tao Jia, Hongbing Tao, Kun Qin, Yulong Wang, Chengkun Liu, Qili Gao

**Affiliations:** School of Remote Sensing and Information Engineering, Wuhan University, 129 Luoyu Road, Wuhan, 430072 China; Tongji Medical College, Huazhong University of Science and Technology, 13 Hangkong Road, Wuhan, 430030 China

**Keywords:** Healthcare center, P-median model, Simulated annealing, Hybrid transportation networks

## Abstract

**Background:**

In a conventional P-median model, demanding points are likely assigned to the closest supplying facilities, but this method exhibits evident limitations in real cases.

**Methods:**

This paper proposed a modified P-median model in which exact and approximate strategies are used. The first strategy aims to enumerate all of the possible combinations of P facilities, and the second strategy adopts simulated annealing to allocate resources considering capacity constraint and spatial compactness constraint. These strategies allow us to choose optimal locations by applying visual analytics, which is rarely employed in location allocation planning.

**Results:**

This model is applied to a case study in Henan Province, China, where three optimal healthcare centers are selected from candidate cities. First, the weighting factor in spatial compactness constraint is visually evaluated to obtain a plausible spatial pattern. Second, three optimal healthcare centers, namely, Zhengzhou, Xinxiang, and Nanyang, are identified in a hybrid transportation network by performing visual analytics. Third, alternative healthcare centers are obtained in a road network and compared with the above solution to understand the impacts of transportation network types.

**Conclusions:**

The optimal healthcare centers are visually detected by employing an improved P-median model, which considers both geographic accessibility and service quality. The optimal solutions are obtained in two transportation networks, which suggest high-speed railways and highways play a significant role respectively.

## Background

A location allocation model generally involves two steps, namely, locating facilities and allocating resources. In the former step, a certain number of facilities are optimally selected from a potential set to provide services; in the latter step, resources are optimally allocated to a set of spatially distributed demanding sites for consumption [1, 2]. Optimality is typically evaluated with an objective function in terms of minimum average travel distance or time, maximum coverage, or minimum cost related to multiple factors. A commonly used model is the P-median model introduced by Hakimi [3]; this model aims to determine the locations of *P* facilities such that the total travel distance from each demanding site to the closest facilities is minimized. In addition, the P-median model is focused on objective function with a maximum coverage [4] or on assignment strategy with a gravity effect [5]. However, the P-median model with an objective function considering spatial compactness cost has been rarely investigated [6].

With a non-trivial role, location allocation analysis is implicated in regional planning and resource allocation for flexibility and refinement [7]; these factors have been extensively investigated and applied in various fields. For instance, studies have been conducted in private facilities to determine optimal locations of warehouses [8] and allocate costs in a hub-spoke telecommunication network [9]. Other studies have proposed an effective configuration of a supply chain network in terms of profit maximization [10]. Moreover, studies on public facilities have mainly focused on deriving optimal deployment of emergency response facilities, such as ambulance sites or fire stations with maximum coverage [11, 12], determining convenient locations for schools to minimize travel distance [13], and addressing problems concerning parking lots [14] or off-street parking facilities [15] in terms of minimum travel distance and maximum demands.

Healthcare centers are categorized as public facilities. In social justice, healthcare centers should be optimally located to improve service accessibility, and medical resources should be reasonably allocated to enhance service quality. Hence, service accessibility in terms of time or distance can be applied to determine the utilization of medical resources [16, 17]. Studies have already adopted accessibility measurement or access-based two-step floating catchment area method to evaluate hospital sites [18–20]. Furthermore, service quality is usually related to healthcare center capacities [21], suggesting that conventional location allocation models in operational research should be modified with a capacity constraint on facilities. This modification undoubtedly increases the computation complexity of a model, and heuristic or meta-heuristic algorithms should be used to cope with this problem [22–25]. However, only a limited number of models have been proposed. For instance, Pirkul and Schilling [26] proposed a lagrangian relaxation method in which covered and uncovered demands are assigned successively. Shariff et al. [21] utilized a modified genetic algorithm that suggests the need for additional new facilities or capacities in existing facilities.

Previous studies on healthcare center location are relied on a homogeneous road network in which each road with the same speed limit or even on a Euclidean plane to determine accessibility measurement in terms of travel time or distance. However, these measurements are slightly different from real situations. No study has considered the effectiveness of the spatial deployment of optimal locations in terms of spatial compactness, although a previous study considered this factor in resource allocation for land development but was limited to a raster space [6]. To the best of our knowledge, only a very limited number of studies have attempted to visually evaluate optimal healthcare center locations by using interactive graphs or plots. Visual analytics can be used to solve complex problems with multiple variables, particularly optimal healthcare center locations with multiple cost variables.

To fill these gaps, we proposed an exact and approximate integrated P-median model that can recommend optimal healthcare center locations from a set of spatially distributed sites. In general, this model is constructed by applying two successive procedures: exact and approximate procedures. In the first procedure, all possible combinations of *P* healthcare centers are enumerated; in the second procedure, a simulated annealing meta-heuristic approach is utilized to allocate medical resources from selected *P* facilities to demanding sites. In this model, a transportation network model is specifically used for the underlying geographic infrastructure. Capacity constraint of healthcare centers and spatial compactness constraint of demanding cities are considered and modeled as cost variables in an objective function. Visual analytics is also applied to help identify optimal locations. Using the proposed method to a real case in China, we aim to answer the following questions. (1) How do we incorporate spatial compactness constraint into our model and further determine its influence on optimal locations of healthcare centers? (2) How do we apply visual analytics to choose optimal healthcare centers with multiple cost variables? (3) How do transportation network types affect optimal healthcare center locations?

The present study has the following structure. In Section 2, the datasets are introduced. In Section 3, the computational framework of a modified P-median model is proposed by considering capacity constraint and spatial compactness constraint. In Section 4, experiment results are presented by applying the proposed method on a case study based on visual analytics. In Section 5, several topics, together with the limitations of the present study, are discussed. In Section 6, conclusions are presented and topics for future studies are proposed.

### Data

Three datasets of the Henan province of China are used in this study. The first dataset is obtained from the Health Department of Henan Province and composed of 38 hospital sites. The second dataset is retrieved from Google Map and consists of 17 cities. The third dataset is obtained from the agency of surveying and mapping and consists of transportation data, including secondary and primary ways, highways, railways, and high-speed railways.

### Hospital sites

**Figure 1:**
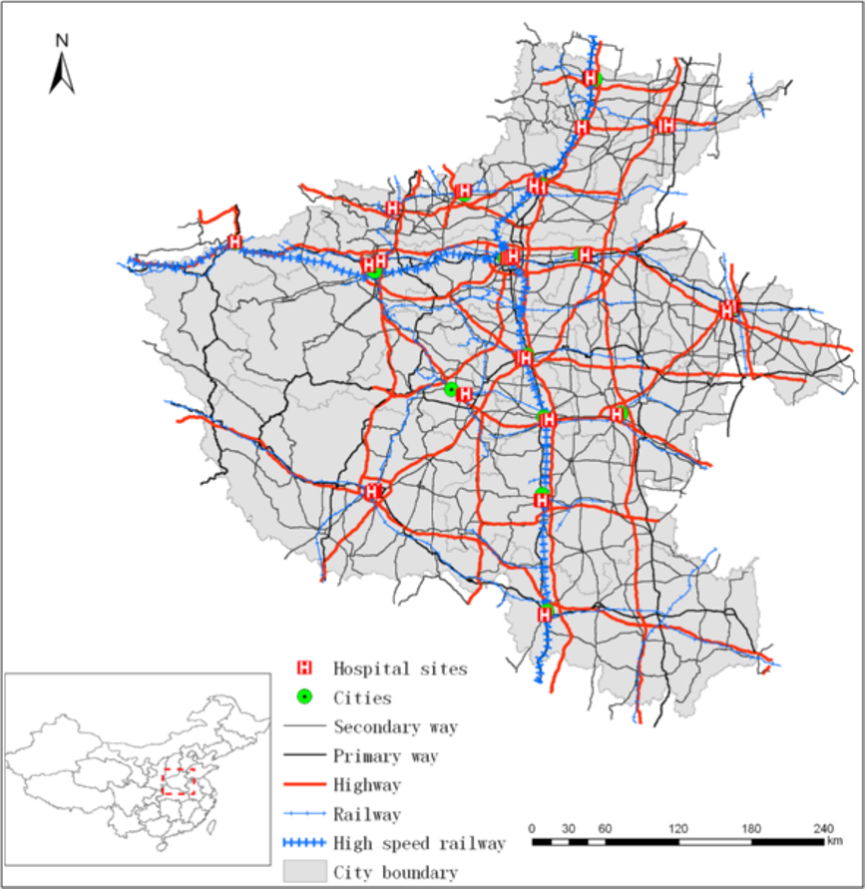
**Map of study area with hospital sites, cities, and transportation infrastructure.**

Thirty-eight tertiary hospitals, which is the highest medical level according to the classification system of Chinese hospitals, are included in the present study. Hospital information includes geographic location in terms of latitude and longitude, hospital name, number of beds, number of key special departments, number of physicians per thousand residents, and number of beds per thousand residents. Geographic information is used to indicate the location of each hospital in the map, as shown in Figure 1 with a red symbol; the number of beds is used to calculate the capacity served by each hospital. The three remaining details are utilized to determine the attractiveness measurement of each hospital (*c.f. Sec. Methodologies*).

### Cities

City data consist of 17 administration centers in Henan Province and include the following information: geographic location in terms of latitude and longitude; city name; and number of residents. Geographic information is used to determine the location of each city in the map, as shown in Figure 1 with a green symbol; the number of residents is utilized to determine medical demands in each city (*c.f. Sec. Methodologies*).

### Transportation data

**Figure 2:**
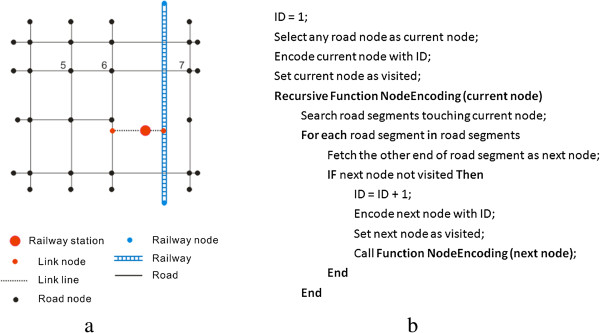
**Illustration of (a) building topological relationship for a synthetic transportation network and (b) its pseudo-code.**

Transportation data are retrieved from the agency of surveying and mapping of Henan Province, and these data include 29,213 road segments with a total length of 40,477 km. These data also include road name and type. Road type is classified into five categories, namely, secondary way, primary way, highway, railway, and high-speed railway. These categories are shown in Figure 1 with different line styles. Moreover, different categories of road types help obtain the speed limit imposed on these roads, and speed limit can be further used to calculate the approximate travel time on each road segment. Table 1 shows that 42.1% of road length is covered by secondary way and only 2.3% of road length is covered by high-speed railway.The topological relationship in a transportation network is essential for navigation-based algorithms, such as Dijkstra algorithm, to calculate the shortest path. However, the road segments in the transportation data are not topologically related in terms of their connection. Moreover, the railway network is not actually connected with the road network. Hence, two steps are conducted to build the topological relationship of the transportation network. First, the railway station is used to find the nearest road segment and railway segment; two link lines are then drawn to connect the railway station to the nearest road segment and railway segment, respectively. This connection is evidently shown by a black dashed line (Figure 2a). Second, the connectivity relationship among the transportation segments is constructed by assigning an ID number to the corresponding origin and destination nodes. This relationship is shown in Figure 2b in which a recursive function is devised to encode the node starting from ID number 1. Moreover, the travel time in each transportation segment is calculated by dividing the segment length by the speed limit. A hybrid transportation network containing a road network and a railway network suitable for navigation are thereby obtained.

**Table 1:** **Proportion of road length with respect to five road categories**

	Secondary way	Primary way	Highway	Railway	High-speed railway
Percentage	42.1%	13.0%	31.1%	10.5%	2.3%
Speed limit	80	100	120	120	300

(Note: speed limit is in kilometers per hour).

### Methodologies

In this section, the methodologies adopted in the present study are described. First, metrics of cities and healthcare centers are elaborated. Second, the basic principle of the P-median model is introduced. Third, a modified P-median model proposed in the present study is illustrated by adopting a meta-heuristic approach of simulated annealing to allocate resources and by accounting for capacity constraint and spatial compactness constraint.

### Metrics of cities and healthcare centers

In China, a healthcare center is an institution or integration of some medical institutions that is to treat the patients with major complex diseases and to train the medical personnel for other hospitals within a certain area. It has the highest medical level in a certain area, and hence selection of potential healthcare centers is very important for the provision and utilization of medical services. For each healthcare center, attribute information is determined by summation of corresponding hospital data; its spatial information is assigned by the location of the corresponding city. Specifically, three metrics including demands of cities, capacity of a healthcare center, and attractiveness of a healthcare center are derived using the following techniques.

### Demands of cities

In the present study, city demand is defined as the number of potential patients with complex diseases that should be transferred to a healthcare center for treatment. To obtain this value, we consider three variables, namely, the number of residents [*N*_*pop*_(*city*)], the average rate of hospitalization (*α*), and the incidence of complex diseases (*θ*). Note that the variable *θ* represents the proportion of patients who should be transferred to healthcare centers. The value of *α* is set as 0.096 and the value of *θ* is set as 0.1. This value can be approximated by the product of these three variables, as shown in Eq. [1]. Using this formula, we obtained city demands.
1

### Capacity of a healthcare center

In the present study, healthcare center capacity is defined as the number of residents that can be served by a healthcare center. To determine this value, we consider two variables, namely, the number of available beds (*N*_*beds*_*(hc)*) and the average length of stay in a hospital (*AlOS*). Note that the value of *AlOS* is set as 10.4 based on a survey in Henan province. This value can be calculated using Eq. [2]. Using this equation, we calculated the capacities of healthcare centers.
2

### Attractiveness of a healthcare center

In the present study, healthcare center attractiveness is defined as the degree of strength of medical service provision. A large value of healthcare center attractiveness suggests that numerous residents prefer to visit this center. In general, healthcare center attractiveness can be determined by combining three variables, namely, the number of key special departments (*N*_*ksd*_), the number of physicians per thousand residents (*N*_*ptr*_), and the number of beds per thousand residents (*N*_*btr*_). To calculate the weight of each variable, we resort to the method of analytic hierarchy process (AHP) [27]. The AHP method is very simple and can be applied using the following steps. (1) A hierarchy of the complex problem at hand is built by decomposing this problem into easily comprehended sub-problems or elements; in the present study, two levels of hierarchy are established in which the first level corresponds to the problem and the second level corresponds to the three elements (variables). (2) Ten experts are invited to independently judge the relative importance of any two elements, and a (3 × 3) matrix is generated by averaging the judgment matrices of these experts. (3) An eigenvector with a maximum eigenvalue is derived, and each component in the eigenvector represents the weight of the corresponding variable. Healthcare center attractiveness value is expressed as Eq. [3], where *Norm()* represents a normalization function to generate the value within [0, 1], and *w*_*1*_, *w*_*2*_, and *w*_*3*_ are the weights of the three variables with values equal to 0.62, 0.252, and 0.128 respectively.
3

### Principles of P-median model

The discrete P-median model was first proposed by Hakimi in 1964 [3]. Since then, this model has been commonly used for location allocation science. This model aims to find *P* facilities such that the total weighted travel distance from all demanding points to the respective closest facilities are minimal. Travel distance can be based on a plane or on a street network, depending on the purpose of the study. The simplicity of the model confers its easy implementation and formulation with an integer-programming problem as follows.
45678

where *i* and *j* are indexes of demanding points and facilities, respectively; *x*_*ij*_ and *y*_*j*_ are decision variables denoting if demanding point *i* is assigned to facility *j* and if facility *j* is selected; *d*_*ij*_ is the distance between demanding point *i* and facility *j*; *weight_d*_*i*_ is the weight value of demanding point *i*; and *P* is the number of facilities to be selected. Eq. [4] is the objective function to be minimized. Eq. [5] is the constraint that requires each demanding point to be assigned to only one facility. Eq. [6] is the constraint ensuring that each demanding point is assigned to a selected facility. Eq. [7] is the constraint ensuring that exact *P* facilities are selected.

Kariv and Hakimi [28] showed that a P-median problem is NP-hard, indicating that this problem can be efficiently solved in polynomial time by a deterministic Turing machine. In some cases, heuristic or meta-heuristic algorithms, such as simulated annealing [24] or genetic algorithm [23], may be utilized to obtain an optimal solution instead of an exact solution. In addition, a conventional P-median model assumes that each demanding point is assigned to the closest facility relaxed by a gravity P-median model [5]. Similarly, studies have relaxed this assumption by assigning demands to the second closest or farther facility if a closer facility exceeds capacity when capacity constraint of facilities is considered [29]. Moreover, the spatial deployment of demanding points to facilities is rarely considered for a discrete P-median model and may have a non-trivial effect on real cases of site location planning [30]. Therefore, our study proposes a modified P-median model that simulates both capacity constraint and spatial compactness constraint as costs in an objective function of a simulated annealing process.

### A modified P-median model

**Figure 3:**
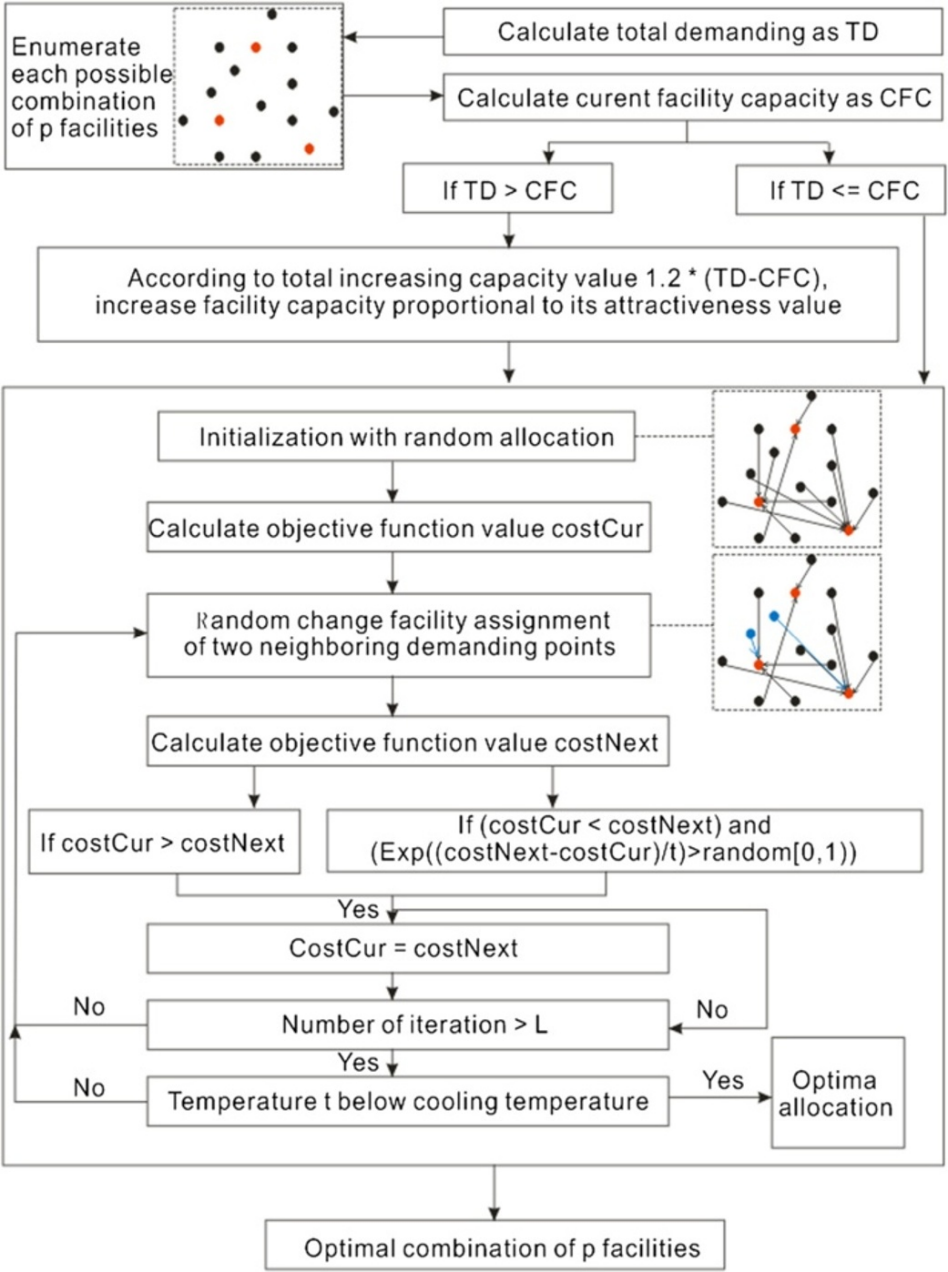
**General flow diagram of computational framework.**

A modified P-median model is elaborated in this section. This model generally adopts exact and approximate strategies to choose optimal locations. In the exact strategy, all combinations of *P* facilities are enumerated; in the approximate strategy, a simulated annealing meta-heuristic approach is used to assign demanding points to facilities. The computational framework is shown in Figure 3 in which a flow diagram illustrates individual steps. Note that the modified P-median model relies completely on the simplicity and elegance of the conventional P-median model. Nonetheless, it enriches the current studies on P-median model by considering both capacity constraint and spatial compactness constraint.

### Alternative P facilities

In this step, all possible combinations of *P* facilities are enumerated. For each combination, the total capacity of the *P* facilities is calculated and compared with the total demanding value. If the *P* facilities cannot serve the total demands, a total increasing capacity is derived as the product of an increasing factor and the difference between capacity and demanding values. Based on the total increasing capacity value, the capacity of each facility is then increased proportionally to its attractiveness value. However, enumeration should be performed considering all possible combinations because of the following points. Location allocation problem is relatively small and a visual analytic strategy helps obtain an optimal solution from potential alternatives with marginal differences.

### Simulated annealing for resource allocation

Kirkpatrick et al. [24] introduced the concept of annealing to combinatorial optimization; this concept is based on the analogy with an annealing process of heated crystals. In the present study, annealing is applied to assign demanding points to respective facilities and functions as follows. First, each demanding point is assigned to a randomly selected facility following the constraints in Eqs. [5, 6 and 7]; thus, current allocation solution is obtained. Second, the objective function in Eq. [4] is used to derive the cost value (*costCur*) of the current allocation solution in which travel time is used instead of travel distance. Third, facility assignment change is performed on two neighboring demanding points that share a common edge in their Voronoi polygons [31]. The next assignment solution is then introduced and the corresponding cost value (*costNext*) is calculated with the same objective function. Fourth, a decision is made either to accept the next assignment solution as the current assignment solution or to reject it. Acceptance is basically determined if the *costCur* value is larger than *costNext* or if the *costCur* value is less than *costNext* while meeting the Metropolis criterion [32] as shown in Eq. [9]. The metropolis criterion allows a movement to a slightly worse solution to avoid being trapped in a local minimum. However, this probability gradually decreases with decreasing temperature *t*. Fifth, steps 3 to 4 are iterated until a maximum number of iterations *L* is reached. Last, once the iteration number exceeds the maximum *L*, temperature *t* is reduced. Steps 3 to 5 are repeated until temperature *t* is below the cooling temperature.
9

However, this meta-heuristic allocation only considers travel time as objective for minimization, which may result in an allocation solution with several facilities overloaded in terms of providing service. In practice, a facility should have capacity constraint in terms of providing service. Therefore, the capacity constraint of a facility is modeled as cost in an objective function, which is explicitly elaborated in the following part.

### Modeling capacity constraint

To model the capacity constraint based on the objective function, we define a variable named *shortageRatio* for each facility, which can be expressed as the fraction of the required capacity value (difference between the value of the current capacity and allocated demands) over the current capacity value. The model shows the extent to which current facilities can satisfy allocated demanding points. A small value of this variable contributes a small cost to the objective function, indicating an optimal location of the current facility in terms of resource allocation under capacity constraint. For instance, a zero value of this variable suggests a facility that can satisfy all of the allocated demands with the current capacity.
10

Moreover, the modified objective function excludes spatial cost in terms of spatial compactness. This constraint is necessary because capacity constraint could force a facility to serve further demanding points, leading to impractical site location and resource allocation.

### Modeling spatial compactness constraint

**Figure 4:**
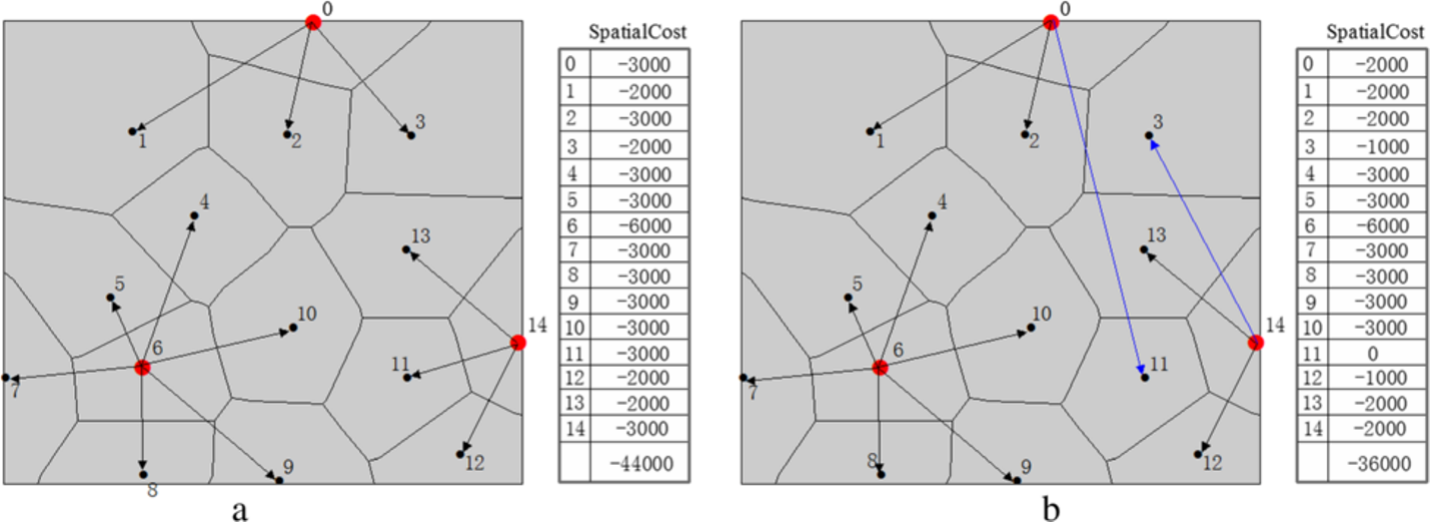
**Illustration of calculating spatial cost with** ***γ*** **=1000 for a synthetic location allocation network in scenario (a) and (b).**

To model the spatial compactness constraint based on the objective function, we define a variable named *spatialCost* for each demanding point; this variable can be described as the product of the number of neighboring demanding points with the same facility assignment and a weighting factor *γ*. This value can be multiplied by -1 to be integrated into the objective function. It reflects the extent to which the compactness or concentration of the current demanding point can contribute to the spatial compactness of a facility-demanding network. A low value of this variable suggests an appropriate location of the current demanding point in terms of spatial deployment of demanding points and likely reduces costs in an objective function. Thus, this variable provides a way to avoid spatial intersections of supplying lines from different facilities and may lead to a plausible planning of the site location and resource allocation. As shown in Figure 4, *spatialCost* is calculated with *γ* =1000 for two different spatial deployments of demanding points in a synthetic network. However, the value of this variable can be adjusted according to *γ*, ultimately affecting optimal solution.

11

### Results and analysis of optimal healthcare centers

In this section, optimal healthcare centers are presented by applying the method to the aforementioned dataset. The results are specifically elaborated regarding three aspects. First, the result of *γ* evaluated under spatial compactness constraint is presented. Second, the potential optimal healthcare centers are shown on the basis of multiple cost variables. Last, the result as to how the properties of transportation network affect optimal healthcare center locations is presented.

### Weighting factor γ of spatial compactness constraint

**Figure 5:**
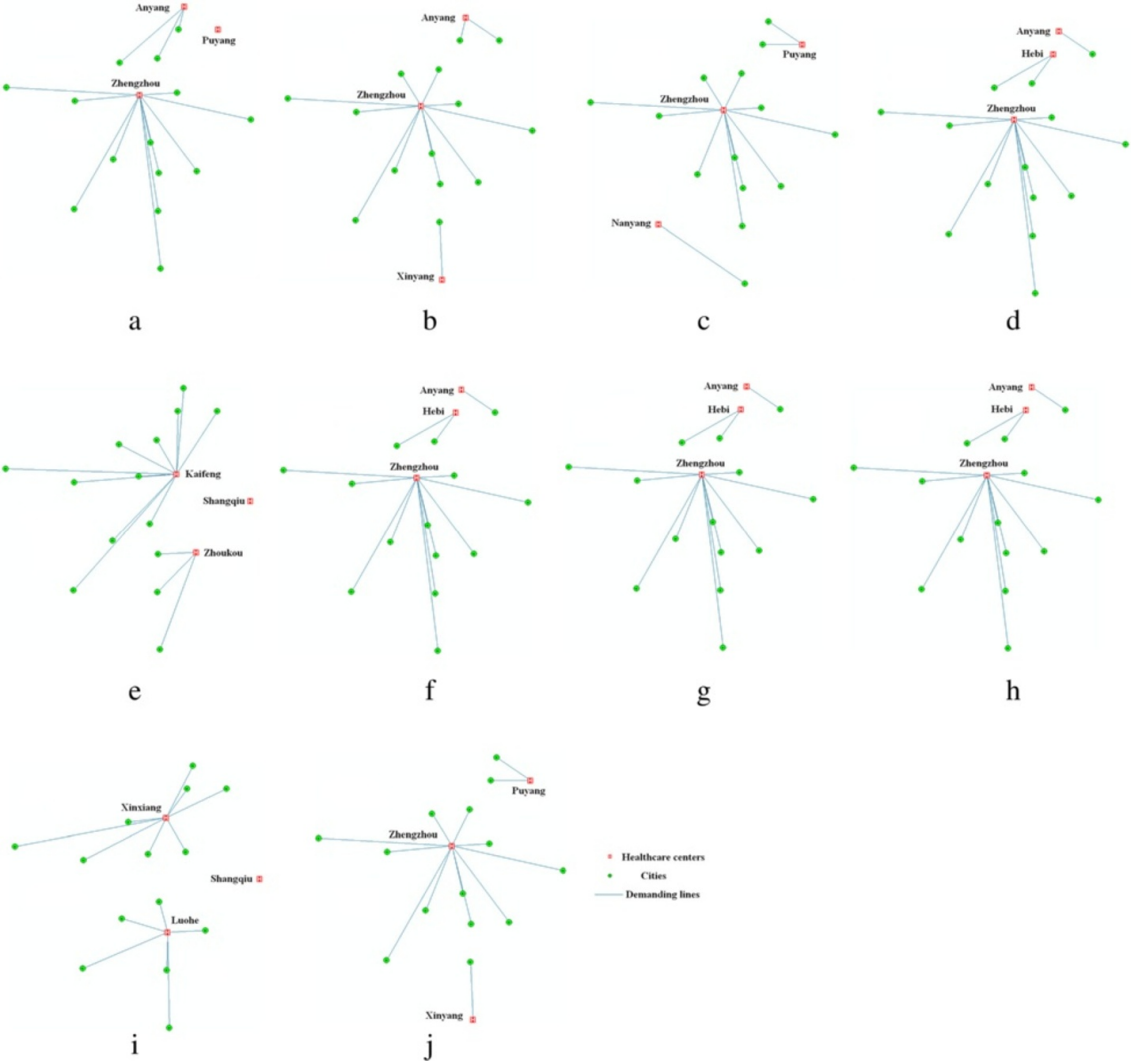
**Spatial deployment patterns of healthcare centers with minimum spatial cost in terms of different** ***γ*** **values: (a) 200, (b) 400, (c) 600, (d) 800, (e) 1000, (f) 1200, (g) 1400, (h) 1600, (i) 1800, (j) 2000.**

Spatial compactness constraint is modeled as a spatial cost in the objective function and affected by *γ*. Spatial cost gradually decreases as *γ* increases. Thus, γ should be selected such that a plausible spatial deployment of demanding cities and supplying healthcare centers can be achieved. To tackle this issue, we show spatial deployment patterns with a minimum spatial cost in terms of different *γ*. In Figure 5, no spatial intersections of supplying lines from different healthcare centers are generally found. For the cities, Zhengzhou, the capital city of Henan Province, is selected as one of the three healthcare centers in most cases. This selection is not surprising because this city has the largest medical resource capacity and lies in the transportation hub. For the spatial distribution of the healthcare centers, healthcare centers with γ = 600 are evenly distributed in space, thereby preventing inequity in geography. In addition, this value provides a local minimum of the sum of travel and capacity costs.

### Optimal healthcare centers on account of multiple cost variables

Once *γ* is determined, optimal healthcare centers with a minimum value in the objective function can be selected. However, the result obtained in this manner suffers from a non-trivial deficiency, indicating that healthcare centers with minimum cost are not necessarily superior to those with the second minimum cost. For instance, decision makers would likely trade-off between the minimum cost and the spatial deployment pattern to choose optimal healthcare centers to avoid geographic inequity. To overcome this deficiency, we adopt an explorative visual analytic technique and vividly present the alternative healthcare centers, and decision makers are provided with a number of graphic interfaces to choose optimal solutions.

**Figure 6:**
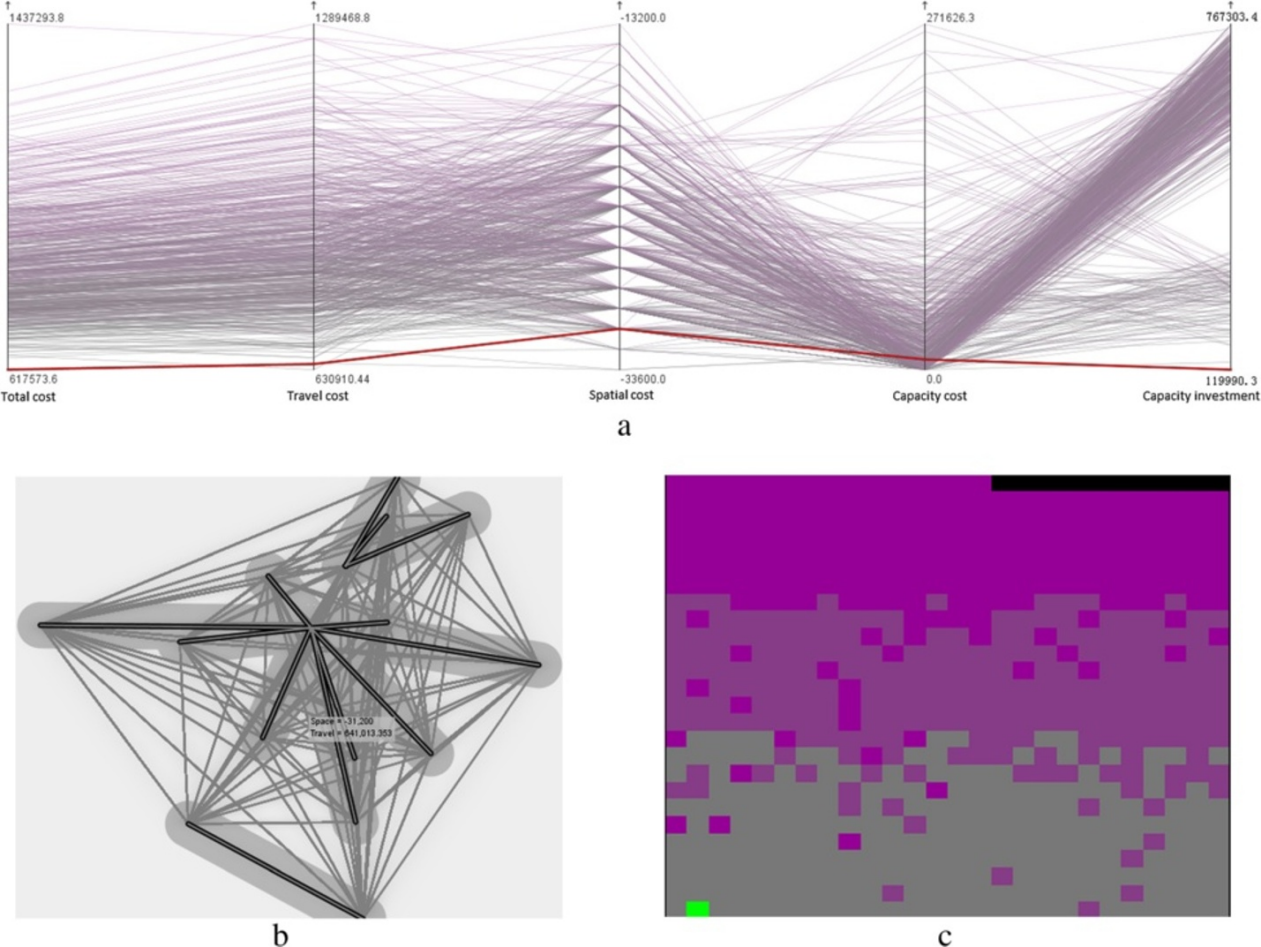
**Choosing optimal healthcare centers in a hybrid transportation network with (a) parallel coordinate plot, (b) bivariate geomap, and (c) space filling raster.** (Note: Two variables, namely, total cost and travel cost, are visualized in space filling raster. Total cost is represented with a color ramp from gray to purple and travel cost is ascendingly sorted with a scan line from left to right and bottom to top.).

**Figure 7:**
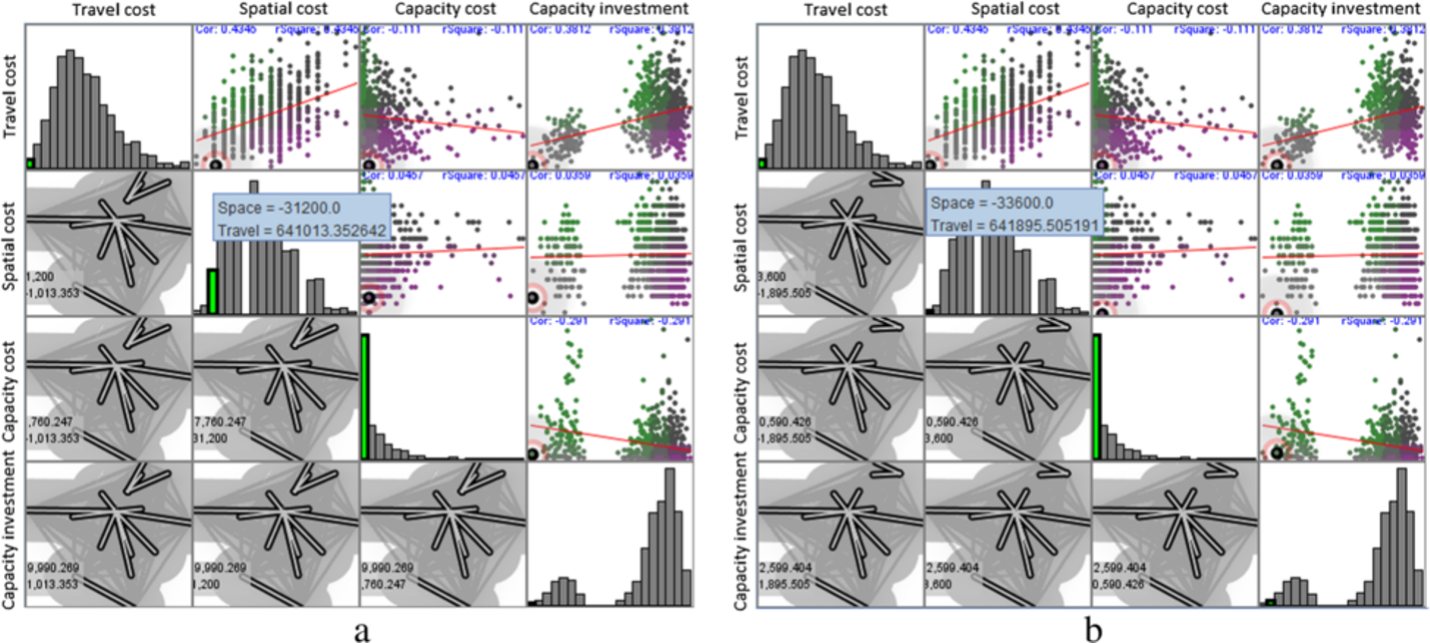
**Visual comparison between two competitive solutions highlighted with large dots: (a) solution with the minimum total cost and (b) solution with minimum spatial cost.** (Note: This is a map and scatter plot matrix of the four variables. Elements in the upper triangular matrix denote the scatter plots of any two variables, and we found that travel cost gradually increases as spatial cost increases or capacity cost decreases. Diagonal entries in the matrix show the histograms of the corresponding variables, and capacity cost can be approximated with a power law-like distribution. Other variables follow a normal or mixed-normal distribution. Elements in the lower triangular matrix are the spatial deployments of any selected solution).

In the present study, the GeoViz Toolkit developed by Frank Hardisty [33] is utilized to help select optimal healthcare centers. This toolkit provides a geovisualization environment to allow users to utilize coordinated exploratory and analytical tools to investigate the geographic data with multiple variables. In the present study, three exploratory visualization tools, namely, parallel coordinate plot, bivariate geomap, and space filling raster, are applied to the dataset containing all of the possible combinations of the three healthcare centers. Each combination is considered as an observation with values corresponding to the five aforementioned variables. Figure 6a shows that each parallel line connects the five variables and it can be highlighted to show the corresponding values. The line with the minimum total cost is specifically highlighted as red for a visual analytic and simultaneously reflected in the two other tools. The bivariate geomap shown in Figure 6b displays the spatial deployment of the healthcare centers and cities with bold demanding lines, indicating a plausible spatial pattern. In addition, the space filling raster shown in Figure 6c identifies this solution with a green pixel, suggesting the minimum value of the total cost and the second minimum value of the travel cost.Three healthcare centers, namely, Zhengzhou, Xinxiang, and Nanyang, are suggested as optimal solutions with a visual analytic procedure. This solution significantly resembles the previous solution elaborated in Section 4.1, although Xinyang instead of Puyang is considered as a healthcare center. To further understand the difference between the two similar solutions, we use a map and scatter plot matrix with four variables (Figure 7), in which the two solutions are symbolized with large dots. The two solutions have relatively small travel, spatial, and capacity costs and capacity investment. These results are represented by the large dots in the lower left corner of the scatter plots in Figure 7. Compared with the previous solution, the current solution has smaller travel and capacity costs and capacity investment but a larger spatial cost. However, whether the current solution is more effective than the previous solution depends on the situations at hand or the purpose of decision makers.

### Optimal healthcare centers in a road network and their transportation usage

**Figure 8:**
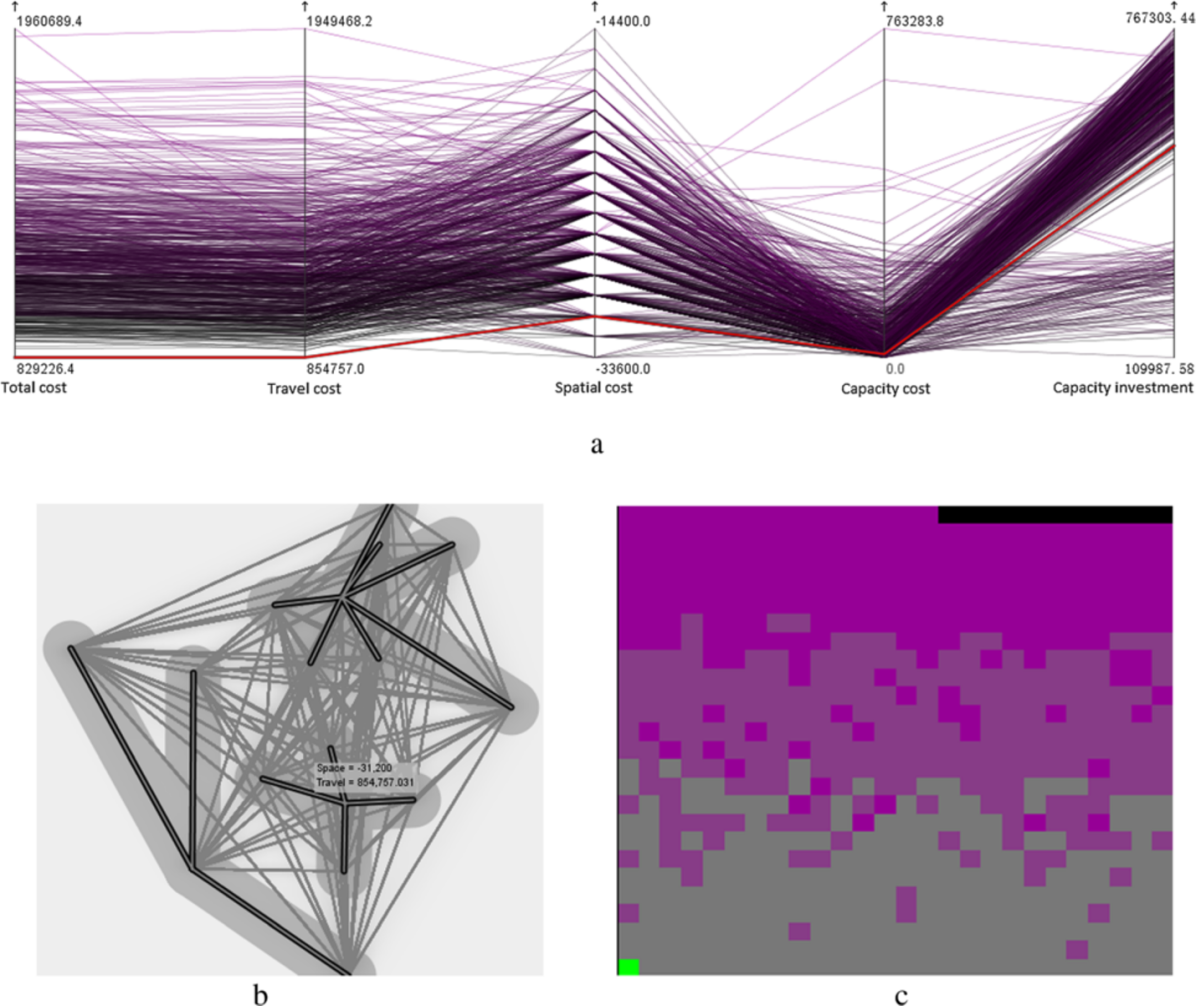
**Identification of optimal healthcare centers in a road network highlighted as (a) a red line in parallel coordinate plot, (b) bold demanding lines in bivariate geomap, and (c) a green pixel in space filling raster.**

**Figure 9:**
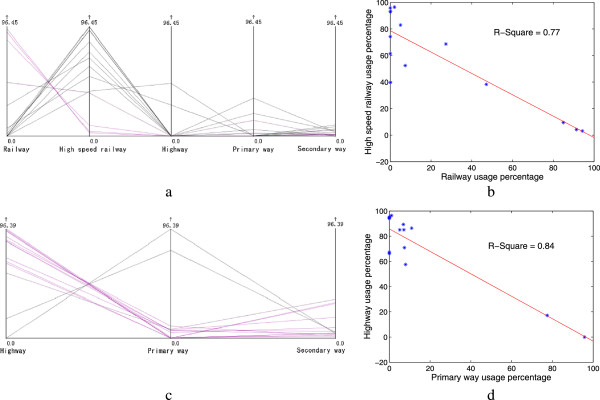
**Transportation usage according to different means of transportation in (a) and (b) a hybrid transportation network and (c) and (d) a road network.**

We have reported the results after we select the optimal healthcare centers in a hybrid transportation network comprising roads and railways. However, we did not delve into issues as to how the optimal healthcare centers can be affected by the types of transportation network and consequently how they utilize the transportation to provide services. With comparative analysis, we derive the optimal healthcare centers for a road network by excluding the railway network. Figure 8 shows that the optimal healthcare centers are identified using the solution with the minimum total cost. This solution includes the healthcare centers of Xinxiang, Nanyang, and Luohe, and it exhibits a plausible spatial pattern and reasonable medical resource allocation among the demanding cities. However, Luohe, instead of Zhengzhou, has been suggested as a healthcare center compared with the solution for the hybrid transportation network, thereby undoubtedly increasing the inputs of the medical resources as seen the high value of capacity investment in Figure 8(a). In addition, the travel cost is increased by approximately 33.2%, indicating that railway network is highly utilized by residents traveling to healthcare centers.To quantitatively understand the transportation usage in the two scenarios, we calculate the proportion of the travel length with respect to different means of transportation in each supplying route. Five means of transportation are utilized in the hybrid transportation network, and these include the railway, high-speed railway, highway, primary way, and secondary way; three means of transportation are used in the road network encompassing only the three latter means of transportation. Figure 9a shows that high-speed railways are extensively used compared with the other means of transportation, and account for approximately 58% of the travel length of all supplying routes. The usage of a high-speed railway is inversely proportional to the usage of railway (Figure 9b). In a road network, the highway is more often used than the two other means of transportation and accounts for an average usage of 72% (Figure 9c). Similarly, highway usage is inversely proportional to the usage of primary way (Figure 9d). These findings suggest that people are more likely to utilize faster available means of transportation to travel.

## Discussions

**Figure 10:**
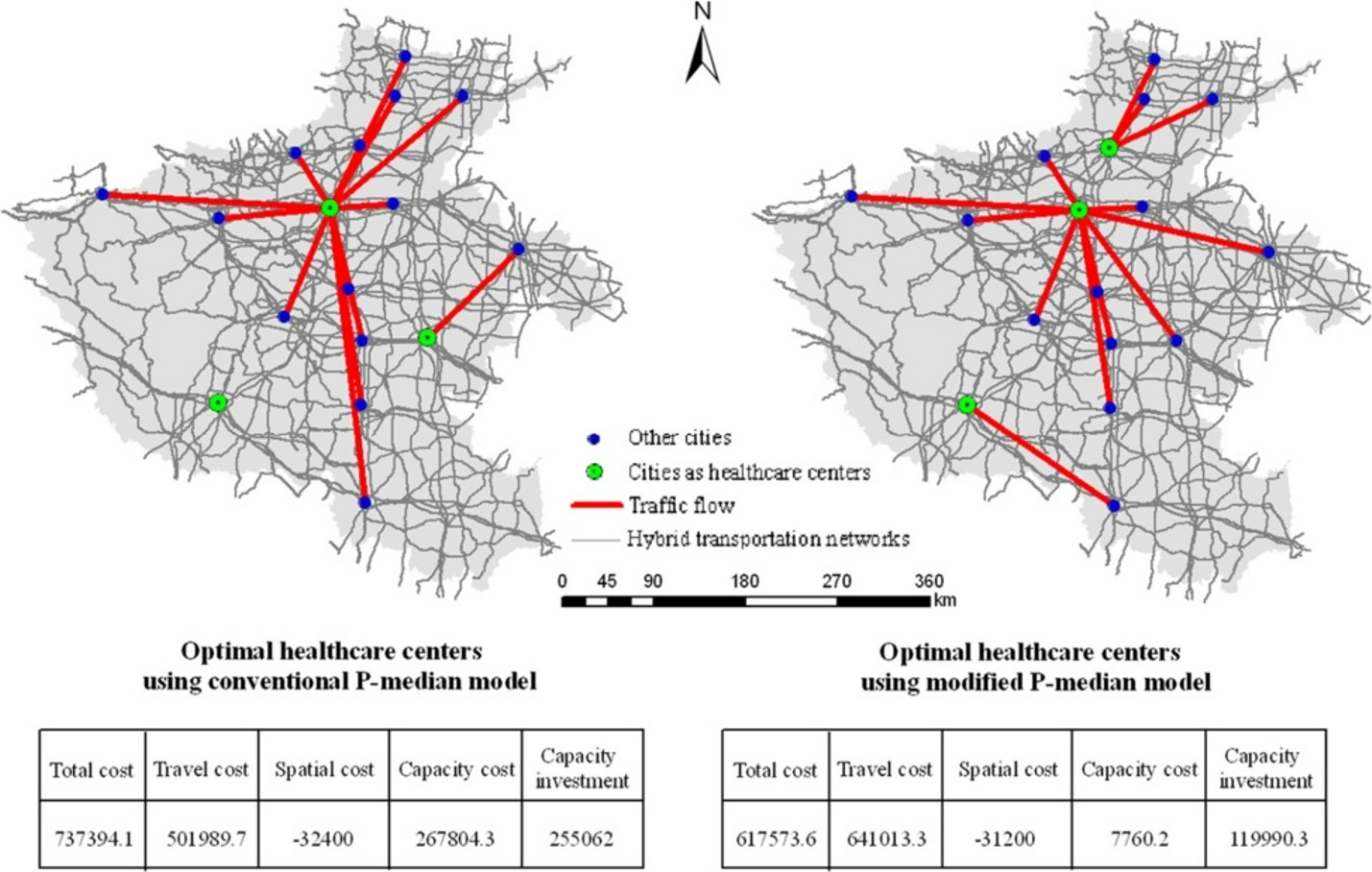
**Comparative results between the solution with the conventional P-median model and the one with the modified P-median model.**

First, the modified P-median model proposed in the present study should be discussed. This model is based on a conventional model by incorporating two procedures. In the first procedure, the capacity constraint imposed on each facility point is modeled and this model assumes that each demanding point is not necessarily assigned to the closest facility. In the second procedure, the spatial compactness constraint imposed on the assignment of demanding point to the facility is modeled, and this model can improve the geographic equity. A γ is utilized to calculate spatial cost, which reflects the degree of spatial compactness. A larger *γ* suggests a larger degree of spatial compactness. The present study adopts an exploratory analysis to specifically determine *γ*, although studies have reported that different solutions may be derived with the same *γ* because of the fuzziness of simulated annealing meta-heuristic approach. Moreover, the result obtained from this model is a series of potential solutions that allows visual analysis to select an optimal solution. However, the applications of this model are narrowed into problems with small sizes and are impractical for a large problem.Second, the modified P-median model is used to solve a real location allocation problem in terms of choosing optimal healthcare centers, which should perform better than the conventional P-median model. To verify this statement, we compared the solution from the conventional P-median model to the one from the modified P-median model. As shown in the following Figure 10, we find that the optimal healthcare centers using the modified P-median model are more reliable and plausible than the other solution. First of all, the solution with our approach displays a better spatial deployment pattern, which can be seen from a smaller value of spatial compactness cost. Then, it suggests a better medical service quality, which can be observed from the smaller values of both capacity cost and capacity investment. In the last, the value of total cost is less than the one from the conventional method, although it has a larger value of travel cost.

Third, geographic factors in general or transportation networks in particular are known to impose a large influence on the determination of the optimal locations of facilities in terms of spatial accessibility. A homogeneous transportation network with a unique speed limit likely results in an even spatial distribution of facilities whereas a hybrid transportation network with various speed limits possibly leads to an uneven distribution of facilities in space. The present study reports that high-speed railways in the hybrid transportation network are highly utilized and serve a significant role in aggregating the demanding cities along the railway to the same healthcare centers, such as Zhengzhou. This finding presents a different pattern from that in a road network, in which highways are extensively used for traveling. However, in reality, residents in demanding cities have diverse choices for transportation types utilized for traveling. Therefore, studies have yet to determine a method to simulate travel behavior of residents in demanding cities and incorporate this behavior into a classic location allocation model. Besides, to enhance the reliability of our model, other factors, such as, natural environment factors, built environment factors, and policy environment factors, should also be considered in the future work.

Fourth, the result obtained from a visual analytic perspective could allow decision makers to select the optimal healthcare centers according to the situations at hand or specific purposes. Visual analytic has been applied to solve complex problems with multiple variables in numerous domains, such as crisis management [34] and social network analysis [35]. To the best of our knowledge, this work presents a benchmark study by applying this technique to the problem of location allocation. In the present study, three healthcare centers, namely, Zhengzhou, Xinxiang, and Nanyang are considered optimal. First of all, the three healthcare centers exhibit a relatively even spatial distribution. For instance, Xinxiang is located in the north of Yellow River, Zhengzhou is at the heart of the province, and Nanyang is found in the south of the study area. Then, the three healthcare centers have the largest amount of medical resources compared with the other solutions, and need only an additional 14% of the medical resources to satisfy such demands. Last, as shown in Table 2, compared with other cities, the three healthcare centers can provide more than 40% of the total medical services but occupy around 26% of the entire population. However, whether the above solution can be accepted is determined by decision makers because they can utilize a visual analytic technique to choose an alternative solution.

**Table 2:** **Comparative results between optimal cities and other cities**

	Residents	Available beds	Key special departments	Physicians
Optimal cities	26.6%	45.0%	55.8%	42.4%
Other cities	73.4%	55.0%	44.2%	57.6%

## Conclusions

This study focused on the problem of locating three healthcare centers in Henan Province, China. We demonstrated that optimal healthcare centers should be located for spatial accessibility, enhanced service, and plausible spatial pattern. Thereafter, a modified P-median model was proposed; this model applies a meta-heuristic simulated annealing to allocate medical resources to minimize total travel, capacity, and spatial costs. The capacity cost is modeled on the basis of the deviation from supplying medical resources to demanding medical resources; hence, a smaller capacity cost likely corresponds to enhanced medical service. In addition, spatial cost is modeled on the basis of the compactness of the spatial deployment of demanding cities, and a small spatial cost could avoid the intersections of supplying lines. Moreover, we measured the value of capacity investment on each solution, and a large value leads to an impractical solution.

This study iterated each candidate in the solution space; thus, visual analytic can be used to evaluate cost variables. Our results suggested that γ in spatial compactness constraint could be vividly determined to obtain a plausible spatial pattern of the optimal healthcare centers. Three cities, namely, Zhengzhou, Xinxiang, and Nanyang, are suggested as optimal healthcare centers, and required lower travel cost, a smaller capacity cost, and a relatively even spatial distribution. Last, the locations of the optimal healthcare centers in two scenarios, namely, a hybrid transportation network and a road network, are visually compared. The results suggest that high-speed railways and highways are highly utilized in the two scenarios and that the solution in the former scenario outperforms that in the latter scenario. However, our model did not consider the impacts from other geographic factors, such as terrain and human behavior, which are points for future studies.
